# Small bowel evisceration in a perforated uterine prolapse

**DOI:** 10.4314/gmj.v55i2.10

**Published:** 2021-06

**Authors:** Eric Y Amakpa, Gertrudis A Hernandez-Gonzalez, Edith Camejo-Rodriguez

**Affiliations:** 1 Ho Teaching Hospital, Department of Obstetrics and Gynecology, Ho. Volta Region, Ghana; 2 University of Health and Allied Sciences, School of Medicine, Department of Internal Medicine and Therapeutics, Ho. Volta Region, Ghana

**Keywords:** Bowel evisceration, uterine prolapse, surgical emergency

## Abstract

The evisceration of the bowel through the vaginal vault is an extremely rare condition and a surgical emergency with a high-reported mortality rate. Vaginal evisceration most commonly affects menopausal women with a hysterectomy or those with previous vaginal surgery. The most common risk factors include the triad of post-menopausal atrophy, previous vaginal surgery and enterocele. Estrogen deficiency in post-menopausal women leads to weaker pelvic support structures and a thin, atrophic vagina, making it more prone to rupture. Previous vaginal surgery leaves scar tissue with diminished vascularity in the vaginal wall and apex, predisposing it to dehiscence. Post hysterectomy, the axis of the vagina may be changed, making it more vertical or shortened and resulting in the vagina losing its valve-like mechanism. We present a 70-year-old female brought to the emergency department with a vaginal prolapse complicated by bowel evisceration, without any history of vaginal surgery, hysterectomy or trauma. The bowel was inspected and irrigated copiously, then reduced into the abdominal cavity as it was still viable. A total vaginal hysterectomy with an anterior and posterior colporrhaphy was done. The patient had a successful recovery with no complication. We present this case due to its rarity, the absence of previous vaginal surgery, trauma, or hysterectomy and the successful multidisciplinary surgical approach with total recovery.

**Funding:**

None

## Introduction

Vaginal vault evisceration is the disruption of the vaginal vault or apex with extrusion of intraperitoneal contents. Vaginal cuff dehiscence is defined as full-thickness separation, partial, or total, of the anterior and posterior edges of the vaginal cuff, but without extrusion. Vault dehiscence predisposes to evisceration.^1^ Vaginal vault evisceration is a rare but serious postoperative complication of hysterectomy. A review on the topic^2^ reported an incidence of 0.032% after pelvic surgery in an observational study performed at the Mayo Clinic. The review also described an incidence of 0.28% and 0.09% of vaginal evisceration after hysterectomy, and an incidence of 0.14% and 4.9% of vaginal cuff dehiscence after total laparoscopic hysterectomy. Vaginal vault evisceration is a surgical emergency necessitating prompt resuscitation and surgical intervention. It leads to significant morbidity and mortality if diagnosis and treatment are delayed. Enterocele and vault prolapse have been described as risk factors of vaginal vault evisceration.^2^

Pelvic organ prolapse (POP) is the descent of 1 or more aspects of the vagina and uterus: the anterior vaginal wall, posterior vaginal wall, the uterus (cervix), or the apex of the vagina (vaginal vault or cuff scar after hysterectomy). This allows nearby organs to herniate into the vaginal space, commonly referred to as cystocele, rectocele, or enterocele.^3^ POP affects around 40% of female.^4^ The prevalence of POP is highly varied according to different studies and is found to be anywhere between 3% and 50%.^5^-^7^ These wide variations are due to differences in study design, inclusion criteria, and accompanying indicator symptoms used among studies. Studies based on telephone surveys without a gynaecological examination rely on the subjective bulge sensation reported by women and estimate the prevalence of POP to be between 2.9% and 8.3%.^5^,^6^ In contrast, in other studies based on an objective gynaecological examination with no regard to women's subjective symptoms, the prevalence of any POP is reported to as high as 50%.^8^ In 2008, a rural study of 174 women in Ghana reported 12.07% of women with POP, 81 % of them were symptomatic, but only 35.3 % had sought treatment because of financial constraints.^9^ The Pelvic Organ Prolapse Quantification System (POP-Q) has classified the POP in five stages:^10^

Stage 0: No prolapse is demonstrated.
Stage 1: The most distal portion of the prolapse is more than 1 cm above the level of the hymen.
Stage 2: The most distal portion of the prolapse is 1 cm or less proximal or distal to the hymenal plane.
Stage 3: The most distal portion of the prolapse protrudes more than 1 cm below the hymen but no farther than 2 cm less than the total vaginal length (for example, not all of the vagina has prolapsed).
Stage 4: Vaginal eversion is essentially complete.

The exact prevalence of uterine prolapse is unknown. The Oxford Family Planning Association study in the United Kingdom followed more than 17 000 women aged 25-39, the annual incidence of hospital admission with prolapse was 20.4/10 000 person-years of observation, and the annual incidence of surgery for prolapse was 16.2/10 000.^11^ Many studies do not distinguish between prolapse of all pelvic organs and prolapse of the uterus alone, which makes it difficult to determine the true incidence.^12^

The aetiology of pelvic organ prolapse is multifactorial. The pelvic organ support study found age to be a risk factor for pelvic organ prolapse; the risk doubled with each decade of life.^13^ Increasing parity was also associated with increasing severity of prolapse. Although vaginal delivery is associated, specific obstetric risk factors remain controversial. Macrosomia, the prolonged second stage of labour, episiotomy, anal sphincter injury, epidural analgesia, and the use of forceps and oxytocin have all been proposed as risk factors but have not been proved.^12^

## Case Report

**Figure 1:**
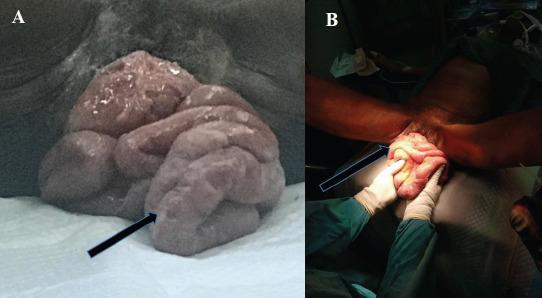
The evisceration of the small bowel (black arrows) through the necrotic vaginal tissue. **A)** After arrival at the emergency department. **B)** Bowel inspection in the theatre

A 70-year-old female presented to the emergency department with acute onset of severe lower abdominal pain, fever 38.5°C with urinary symptoms, and acute evisceration of her bowels through her vagina. She had a history of four vaginal deliveries, and few months after her last delivery, 28 years ago, she noticed a mass *per vaginum*. The mass increased progressively over the period, initially brought on straining and subsequently an overt mass through the vagina with necrotic patches (Figure 1-A), which she was dressing at home. She did not seek medical intervention and used herbal medication, but two years ago, she became bedridden after the mass was big enough to impede ambulation. On the day before presentation, her bowels abruptly gushed out as she sat down to dress the necrotic areas.

### Diagnostic Assessment

On arrival to the emergency department, the patient was in severe pain with a fever 38.5°C, but other vital signs were within normal limits. Physical examination was remarkable for moderately dry skin and mucosa, pallor, and lower abdominal tenderness. Limb muscle masses were generally atrophic. About 60cm of small bowel was seen protruding through a perforation in one of several necrotic areas of a stage IV prolapsed uterus. There was an anterior vaginal wall prolapse with the urinary bladder completely prolapsed. Rectal exams were unremarkable.

Intravenous access was secured, and samples were taken for investigation: Full blood count, Random blood sugar, clothing profile, blood urea and electrolytes, creatinine, Hepatitis B and C, and HIV screening, to assess the general status of the patient, hemodynamic, metabolic status and electrolytes for surgery, and to find out the evidence of infection.

She was relieved of pain with pethidine, hydrated adequately and administered broad-spectrum antibiotics. She was prepared for emergency surgery after assessment by the Anesthesia and Gynecological teams; the surgical team was also part of the review and inspection of bowels. Pre-operatively, the eviscerated bowel were covered with wet abdominal towels with normal saline. Intra-operatively, under general anaesthesia, the bowel was inspected (Figure 1-B) and irrigated copiously with normal saline and povidone-iodine solution. Resection and repair were done to the necrotic tissues. The bowels were reduced into the abdominal cavity as it was still viable. A total vaginal hysterectomy and an anterior and posterior colporrhaphy were done. Her condition was satisfactory in the immediate postoperative period. Physiotherapy was initiated on the 3^rd^ postoperative day with a good response. She was discharged home on Day 10.

At follow-up at the out-patient clinic on day 25, the wound had healed well, bowel function was satisfactory, and muscle strength had improved significantly.

## Discussion

Evisceration of the bowel through the vaginal vault is considered a surgical emergency, and it is a rare condition;^4^ less than 100 cases had ever been reported.^14^ In a review of 12 patients by Croak et al., the mean age for evisceration was 62 years and patients usually presented with pain, vaginal bleeding and abdominal pressure.^15^ In our case, the main complaint was an acute onset of severe lower abdominal pain with fever 38.5°C and urinary symptoms. Vaginal evisceration most commonly affects menopausal women, especially those with a hysterectomy and/or previous vaginal surgery.^14^ In premenopausal women, coital trauma, instrumentation or obstetric injury is the predisposing factor. Our patient was an elderly woman who did not have any history of vaginal surgery or hysterectomy but with a long-standing uterine prolapse without medical treatment. The evisceration of the small bowel through the vagina is a surgical and acute gynaecological emergency, and many complications have been reported, including ischemia, abdominal sepsis and deep vein thrombosis (DVT).^12^ A mortality rate of 6% has been reported.^15^ Prompt diagnosis should be made and resuscitation carried out if necessary. A multidisciplinary approach should be taken in the emergency department and general surgeons should be involved at an early stage, if bowel is eviscerated. Once the woman is stable, a full history should be obtained and a thorough physical and pelvic examination should be performed. If bowel is protruding from the vagina, it is crucial to preserve its viability. An attempt should be made to reposition it intraperitoneally, if this fails, following stabilization, prompt arrangements should be made for surgical repair.^2^

Various approaches for treating vaginal evisceration have been proposed: abdominal, vaginal, and laparoscopic, combined abdominal–vaginal or combined laparoscopic–vaginal. The choice of approach depends on several factors: viability of the prolapsed bowel, evidence of a foreign body in the peritoneal cavity that is not accessible through the vaginal route, and the surgeon's ability to replace the prolapsed bowel.^2^ The most common complications of hysterectomy can be categorized as infectious, venous thromboembolic, genitourinary (GU), gastrointestinal (GI) tract injury, bleeding, nerve injury, and vaginal cuff dehiscence.^16^ Considering the association between hysterectomy and Post-Hysterectomy Vault Prolapse (PHVP), some preventive techniques are of value at hysterectomy to reduce that risk, like McCall culdoplasty at the time of vaginal hysterectomy, the suturing of the cardinal and uterosacral ligaments to the vaginal cuff at the time of hysterectomy is also effective following both abdominal and vaginal hysterectomies; the sacrospinous fixation (SSF) at the time of vaginal hysterectomy is another technique which is considered when the vault descends to the introitus during closure.^17^

Our patient was immediately admitted and managed as a surgical emergency involving a multidisciplinary surgical team. The surgical treatment was done by vaginal approach, commonly used when the eviscerated bowel is viable, i.e. pink, with peristalsis, like in our case. It was reduced back into the abdominal cavity through the vagina, and repair attempted vaginally. It was a successful surgical intervention with total postoperative recovery and no complications reported.

We present this case to raise awareness of the management strategies, considering the significant mortality rates associated with this condition. Secondly, it emphasizes the importance of interdisciplinary surgical cooperation to achieve the best possible outcome for patients.

## Conclusion

Evisceration of the bowel through the vaginal vault is an extremely rare surgical emergency.^4^ POP is quite common in post-menopausal women, especially the multiparous.^14^ Once diagnosed, it must be treated adequately to improve quality of life and avoid complications and mortality.
